# Does a patient with acquired arbovirus infection have a hearing impairment? A scoping review of hearing changes in an adult with Dengue, Chikungunya, and Zika^☆^

**DOI:** 10.1016/j.bjorl.2023.101342

**Published:** 2023-10-11

**Authors:** Leonardo Gleygson Angelo Venâncio, Lilian Ferreira Muniz, Lais Cristine Delgado da Hora, Jéssica Dayane da Silva, Gabriela Silva Teixeira Cavalcanti, Mariana de Carvalho Leal, Sílvio da Silva Caldas Neto

**Affiliations:** a Universidade Federal de Pernambuco (UFPE), Recife, PE, Brazil; b Universidade Federal do Rio de Janeiro (UFRJ), Rio de Janeiro, RJ, Brazil

**Keywords:** Zika virus, Chikungunya virus, Dengue, Hearing disorders, Auditory perceptual disorders

## Abstract

**Objectives:**

To identify and understand the evidence regarding hearing changes related to acquired Dengue, Chikungunya, and Zika virus infection in adult individuals.

**Methods:**

A scoping review was performed according to the recommendations of The Joanna Briggs Institute and guidelines of the Preferred Reporting Items for Systematic Reviews and Meta-Analyses extension for Scoping Reviews in the Embase, PubMed/Medline, ScienceDirect, Scopus, and Web of Science databases without restriction on language and year of publication. Case studies, observational studies, and clinical trials reporting hearing loss in adult subjects (>18–60 years of age) of both sexes with DENV, CHIKV, or ZIKV diagnosed by positive molecular/serological examination by RT-PCR or IgM/IgG by ELISA method were included.

**Results:**

Thirteen studies met the inclusion criteria and were selected for review. The occurrence of auditory symptoms caused by arboviroses and the presence of permanent or transient sensorineural hearing loss was variable in adults.

**Conclusions:**

Dengue, Chikungunya, and Zika infections in adults are associated with a variety of auditory symptoms. The frequency of permanent or transient sensorineural hearing loss is low but not negligible.

## Highlights

• Hearing alteration may occur during Dengue, Chikungunya, and Zika infections.
• Otalgia, hypoacusis, vertigo and tinnitus were the most common symptoms.
• Sensorineural hearing loss was more notiaceable in adults exposed to Zika virus.
• The actual effect of arboviruses on hearing from adults is unknown.

## Introduction

Dengue (DENV), Chikungunya (CHIKV), and Zika (ZIKV) are arboviruses of endemic co-circulation in Brazil.^1^ They are considered a public health concern worldwide due to their history of resurgence associated with environmental and social factors that favor their occurrence, especially situations of sanitary and economic vulnerability.^2^, ^3^

The infection caused by these pathogens may result in immediate or late hearing sequelae that affect different age groups because of damage to the structures or functions of the inner ear.^4^, ^5^ In addition, different auditory manifestations have been reported for patients with DENV, such as tinnitus, vertigo, sudden hearing loss, and sound intolerance,^6^ however, these outcomes were heterogeneous, and the sample size was not representative.

As for CHIKV, one study^7^ showed that a 31-year-old adult patient who recovered from infection had hearing loss with persistent auditory symptoms; however, the causal mechanisms were unclear. In contrast, ZIKV is highlighted for its high prevalence and a causal link to fetal and congenital neurological abnormalities that include microcephaly and Guillain-Barré Syndrome (GBS), a rare immune-mediated condition affecting peripheral nerves.^8^

Early evidence^9^ has shown that prenatal exposure to ZIKV infection is associated with sensory-neural hearing loss. In general, its effect on the infant population has been well studied,^10^ and in 2019, the Joint Committee on Infant Hearing inserted prenatal exposure to ZIKV as a risk factor for hearing loss.^11^ This report provided strong evidence of the relationship of ZIKV with early hearing impairment and suggested follow-up beyond the pediatric age group.^11^

However, in adults, the actions of arboviruses on hearing are still poorly understood.^12^ In summary, there are still gaps about the main hearing changes found in adult individuals with DENV, CHIKV, and ZIKV, with only a few reports of sudden deafness after infection.^5^, ^7^, ^13^

Therefore, the aim of this scoping review is to identify and understand the evidence regarding hearing changes related to acquired Dengue, Chikungunya, and Zika virus infection in adult individuals.

## Methods

The literature review was conducted according to the recommendations of The Joanna Briggs Institute (JBI) for scoping reviews^14^ and guidelines of the Preferred Reporting Items for Systematic Reviews and Meta-Analyses (PRISMA 2020).^15^ The complete research protocol was registered and previously published in the International Prospective Register of Systematic Reviews (PROSPERO) under the number CRD42022335879.

### Review question

The guiding research question “What hearing characteristics may be altered in adult individuals with confirmed DENV, CHIKV, and/or ZIKV infection?” was designed for the selection and search of the studies through the Population, Concept, and Context strategy. Thus, “P” was defined as adult patients (>18 years), “C” as hearing characteristics, and the last “C” as an infection acquired by the arboviruses of Dengue, Chikungunya, and Zika.

### Data search

The literature search was conducted up to October 31, 2022, using Embase, PubMed/Medline, ScienceDirect, Scopus, and Web of Science databases. The search strategy was tailored to each database and included descriptors and keywords related to arboviruses and hearing impairment (Supplementary Table 1). No age range limiters were used to ensure the retrieval of as many relevant studies as possible.

### Eligibility criteria

Case studies, observational studies, and clinical trials reporting hearing loss in adult subjects (>18–60 years of age) of both sexes with DENV, CHIKV, or ZIKV diagnosed by positive molecular/serological examination by RT-PCR or IgM/IgG by ELISA method were included. There was no restriction on the year and language of publication.

Studies that included individuals with hearing loss or complaints prior to infection, a history of exposure to constant noise (80 dBNa for more than 8 h/day), psychiatric disorders, and neurological and genetic syndromes, congenital or acquired prior to infection were excluded. In addition to in-vitro studies, animal studies, editorials, book chapters, reports, commentaries, notes, conference abstracts, and literature reviews.

### Study selection

Data analysis occurred in four steps: identification, screening, eligibility, and inclusion. In the identification stage, appropriate studies were selected by individual database searches. The bibliographies of included studies were manually reviewed for additional references.

The reference manager application Rayyan^16^ was used to store and share studies between reviewers and to remove duplicates. In the screening and eligibility step, the title, abstract, and full text were read by two independent reviewers to rule out studies that did not meet the eligibility criteria. Any discrepancies between them on study eligibility were resolved through discussion or after consultation with a third team member. At the inclusion stage, studies that met all the previous steps were aggregated for data extraction.

### Data extraction and analysis

For analysis, information on study identification (author, year, and place), study design, sample characteristics (population, sample size, and age), presence and type of arbovirus, presence of associated neurological manifestation, hearing assessment method and hearing alteration were extracted in a Microsoft Office — Excel® spreadsheet.

The grades of hearing impairment were reclassified to homogenize the results between the studies considering four-frequency Pure-Tone Average (4fPTA) by obtaining the means of the thresholds at 500, 1000, 2000, and 4000 Hz for each ear, and the values suggested by the World Health Organization as follows^17^: (1) normal, ≤19.50; (2) mild, 19.51–34.5; (3) moderate, 34.51–49.5; (4) moderately severe, 49.51–64.5; (5) severe, 64.51–80.5; and (6) profound, ≥80.51 dB HL.

## Results

After searching the databases, 731 references of potential studies were identified, plus two references retrieved by manual search in the citations and references. After removing the duplicates, 335 articles were screened by reading the title and abstract, where 310 articles were excluded for not answering the guiding question of this research.

**Figure 1:**
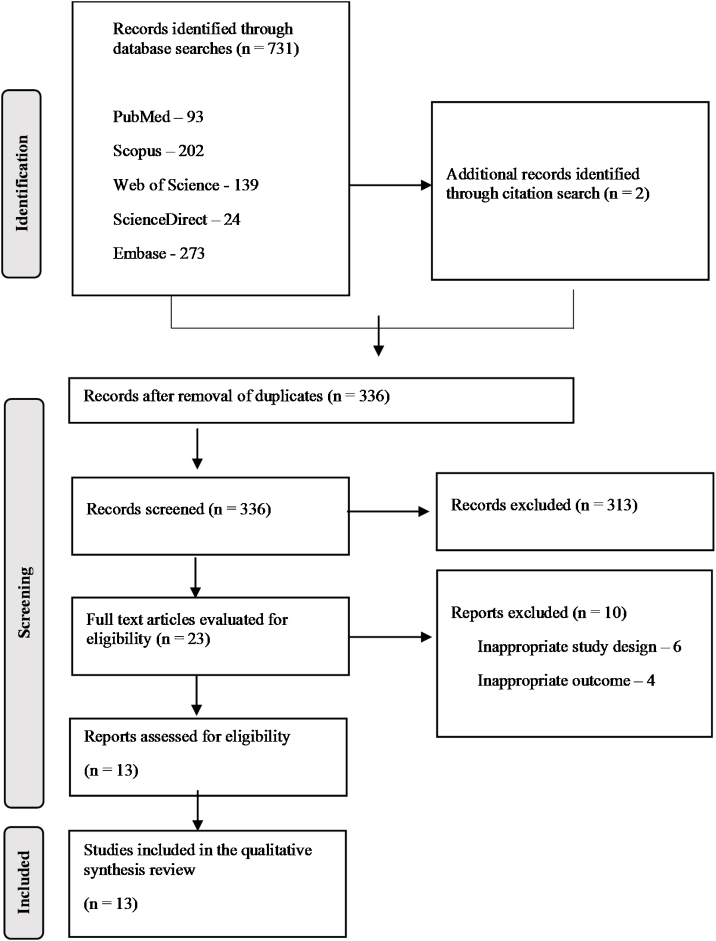
Flow diagram of study selection.

The remaining 25 articles were assessed for eligibility by reading the full text. Of these, six articles were excluded for inappropriate study design and 4 for no auditory outcomes. A detailed overview of the study selection process is presented in the flow chart in Fig. 1.

A description of the identifying characteristics of the 13 included studies is presented in Table 1. Overall, regarding the presence and type of arbovirus, most studied, six studies^5^, ^6^, ^18^, ^19^, ^20^, ^21^ described dengue-related hearing changes in adult subjects.

**Table 1:** Characteristics of the studies included in the review in adult subjects with a confirmed infection by Dengue, Chikungunya, and Zika viruses.

Arbovirus	Author/year	Country	Aim	Study design	Sample (N)	Mean age (years)	Hearing assessment method	Main conclusions
Dengue	Denis et al., 2003	Brazil	Evaluate patients with dengue who present with otorhinolaryngological symptoms	Cross-sectional study	30	33.7	Self-reporting	The clinical suspicion of dengue is essential because of the different otorhinolaryngological manifestations
	Diniz et al., 2021	Brazil	To report a case of aseptic meningitis, acute renal failure, and sensorineural hearing loss in a 42-year-old man with severe dengue fever	Case report	1	42	Audiometry	Six-month follow-up showed persistent deafness, suggesting an association between dengue and hearing loss
	Mughal et al., 2022	Pakistan	To report a case of unilateral sensorineural hearing loss after dengue	Case report	1	46	Rinne test, Webber test and audiometry	Sensory sensorineural hearing loss is a rare presentation in dengue that doctors need to investigate
	Rahme et al., 2020	Brazil	To describe four patients who presented with serologically confirmed dengue infection and cochleovestibular manifestations	Case report	4	55	Audiometry, vídeo head impulse test and acufenometry	The cochleovestibular manifestations in dengue are heterogeneous
	Ribeiro et al., 2015	Brazil	To present a case of dengue hemorrhagic fever that evolved with sensorineural hearing loss	Case report	1	60	Audiometry	No other cause was found for sudden deafness and the correlation with dengue fever was questioned
	Soni et al., 2021	India	To explore the association of dengue with hearing loss	Prospective cohort	10	29	Audiometry, tympanometry, brainstem auditory evoked potential, and steady state	Hearing loss in dengue, even if mild, is irreversible. The cause of the loss has not yet been found, and further studies are needed
Chikungunya	Couturier et al., 2012	France	To measure the frequency and risk factors for rheumatic manifestations after chikungunya infection and to assess their impact on quality of life	Prospective cohort	227	50.3	Questionnaire	Medical follow-up was recommended to support possible associated depression and anxiety
	Dutta et al., 2011	India	Finding the prevalence of chikungunya in Assam, northeast India	Cross-sectional study	10	NR	Audiometry	Highlights the importance of an epidemiological and entomological investigation for detection of the emergence of chikungunya
	Jain et al., 2018	India	Report two cases of chikungunya encephalitis	Case report	2	32.5	NR	Neurological complications may occur during the infectious process or after a period of 15–20 days
Zika	Aspahan et al., 2019	Brazil	To report a case of neuromyelitis optical spectrum disorder associated with Zika virus infection	Case report	1	35	NR	The pathophysiology of neurological disorders related to arbovirus infections has not yet been established, and further research is needed for this purpose
	Martins et al., 2017	Brazil	To characterize the otologic findings in two adult patients, post-infection by Zika virus	Case study series	2	45	Audiometry, tympanometry, brainstem evoked potentials, transient and distortion product evoked otoacoustic emissions	Audiological findings demonstrate possible neuronal involvement in the complaints presented, associated or not with the peripheral component, in infected patients
	Tappe et al., 2015	Germany	To report an acute Zika infection that presented with bilateral hearing difficulties during illness	Case report	1	45	Self-reporting	The cause of hearing difficulties remains unclear. Increased clinical and laboratory awareness may help diagnose outside epidemic events
	Vinhaes et al., 2016	Brazil	Report one confirmed and two probable cases of Zika with transient sensorineural hearing loss	Case report	3	23	Audiometry	An association between Zika infection and transient hearing loss has been suggested

NR, not reported.

The studies were published between the years 2003^18^ and 2022,^20^ and most of them were produced in Brazil (n = 7 articles).^5^, ^6^, ^13^, ^18^^,^^19^, ^22^, ^23^ The most frequent study design was a case report in eight studies.^5^, ^6^, ^12^, ^13^^,^^19^, ^20^, ^22^, ^24^ The sample size and mean age ranged in the studies from 1^5^, ^12^, ^19^, ^20^^,^^22^ to 227^25^ participants and from 23^13^ to 60^5^ years of age.

Two studies mentioned neurological alterations associated with the researched arboviruses, one relating CHIKV^24^ in a case of encephalitis in the brainstem and another pertinent to ZIKV^22^ in a case of acute myelitis. However, the neurological issue was not assessed and was directly associated with the hearing alterations presented.

Furthermore, the most commonly used hearing assessment method was tonal audiometry (n = 8 studies).^5^, ^6^, ^7^, ^13^, ^19^, ^20^, ^21^, ^23^ In contrast, two studies^22^, ^24^ did not clarify which hearing assessment methodology was used to confirm hearing difficulties and the occurrence of hearing loss from arbovirus infections.

### Main hearing alterations in Dengue, Chikungunya, and Zika

The main hearing alterations in adult individuals with a confirmed infection by DENV, CHIKV, and ZIKV viruses are presented in Table 2.

**Table 2:** Main hearing alterations in adult individuals with a confirmed infection by Dengue, Chikungunya, and Zika viruses.

Arbovirus	Study	Hearing results											Change in the grade of hearing loss
		Normal hearing (N)	Symptoms and complaints					Improvement of symptoms	Hearing loss				
			Hypoacusis	Hyperacusis	Otalgia	Vertigo/tinnitus	Tinnitus		(N)	Type	Grade	Ear	
Dengue (n = 47)	Denis et al., 2003	‒	‒	‒	11	6	2	NR	‒	‒	‒	‒	‒
	Diniz et al., 2021	‒	‒	‒	‒	1	‒	NR	1	SN	Moderately severe on right and profound on left	Both	Yes
	Mughal et al., 2022	‒	1	‒	‒	‒	1	Yes	1	SN	Profound	Left	Yes
	Rahme et al., 2020	1	‒	1	‒	2	3	Yes	2	SN	Mild on right and profound on left	Left	Yes
	Ribeiro et al., 2015	‒	‒	‒	‒	‒	‒	‒	1	SN	Profound	Both	No
	Soni et al., 2021	7	2	‒	‒	‒	1	NR	3	SN	Mild (NS)	Both	NS
Chikungunya	Couturier et al., 2012	‒	13	‒	‒	‒	‒	NR	‒	‒	‒	‒	‒
	Dutta et al., 2011	‒	‒	‒	‒	‒	‒	‒	1	SN	Mild to severe (NS)	Both	NS
	Jain et al., 2018	‒	1	‒	‒	‒	‒	NR	‒	‒	‒	‒	‒
Zika	Aspahan et al., 2019	‒	1	‒	‒	1	‒	NR	‒	‒	‒	‒	‒
	Martins et al., 2017	1	‒	‒	‒	2	2	NR	1	SN	Moderate (NS)	Left	NS
	Tappe et al., 2015	‒	1	‒	‒	‒	‒	Yes	‒	‒	‒	‒	‒
	Vinhaes et al., 2016	1	‒	‒	‒	1	2	NR	2	SN	Severe on right and profound on left	Both	Yes

NR, not reported; NS, not specified exact hearing thresholds, so the degree of hearing loss was not reclassified; SN, sensorineural; PM, mixed hearing loss.

Six studies^5^, ^6^, ^18^, ^19^, ^20^, ^21^ investigated hearing changes in 47 individuals with DENV. In these individuals, otalgia was the most frequent symptom (23.40%),^18^ followed by vertigo/tinnitus (19.14%),^6^, ^18^, ^19^ tinnitus (14.89%)^6^, ^18^, ^20^, ^21^ and hypoacusis (6.38%).^20^, ^21^ Symptom improvement was reported in two studies.^6^, ^20^ The occurrence of sensorineural hearing loss was 17.02% (n = 8 individuals)^5^, ^6^, ^19^, ^20^, ^21^ of profound grade^5^, ^6^, ^19^, ^20^ and with significant change in hearing thresholds implying improvement in the degree of hearing loss over time in most studies (Supplementary Table 2).^6^, ^19^, ^20^

Three studies^7^, ^24^, ^25^ investigated hearing changes in 239 individuals with CHIKV. Hypoacusis was the only symptom reported (5.85%),^24^, ^25^ and mild to severe sensory sensorineural hearing loss was reported only in a single study for one individual,^7^ with no significant change in hearing thresholds implying an improvement in the degree of hearing loss.^7^ It is noteworthy that the authors should have specified the tone thresholds at each Frequency.^7^ Unfortunately, this made it impossible to reclassify the degree of hearing loss for homogeneity of the data.

Three studies^12^, ^22^, ^23^ investigated hearing alterations in 7 individuals with ZIKV. The most frequent symptoms were vertigo/tinnitus (57.14%)^13^, ^22^, ^23^ and tinnitus (57.14%).^13^, ^23^ It was not reported whether there was an improvement in these specific symptoms. The occurrence of moderate-grade sensorineural hearing loss was 42.85% (n = 3 individuals)^13^, ^23^ with a change in in hearing thresholds implying improvement in the degree of hearing loss.^13^

## Discussion

This scoping review is the first study of its kind to provide systematic and semiquantitative insight that the presence of hearing alterations during infection with DENV, CHIKV, and ZIKV viruses in adult subjects agrees with previously published findings in other viral infections showing that the auditory system can be compromised to varying degrees of severity.^26^

The high endemic prevalence of dengue, especially in Brazil, where most studies were produced, associated with social and environmental issues, justifies why this has been the most studied arbovirus and is even related to hearing alterations.^27^, ^28^

An audiometry may have been the most widely used hearing evaluation method because it reveals the integrity of the peripheral auditory pathways besides accurately estimating hearing thresholds. However, since damage to the central nervous system has already been reported in adult individuals with ZIKV or CHIKV.^29^, ^30^ Assessment of central auditory processing was expected; however, no study has applied tests for this purpose, demonstrating that there are gaps in central auditory functioning in the adult population.

The heterogeneity of hearing alterations in arboviruses is a common finding,^26^ in which sensory sensorineural hearing loss can occur with distinct degrees of severity and have transient or permanent characteristic degrees.^5^, ^7^, ^13^ DENV is not recognized as causing hearing loss,^5^ and its pathological mechanisms have not been clarified.^21^ However, the most accepted hypothesis points out that hearing loss occurs by the impairment in vascular permeability of the terminal artery that supplies the cochlea due to the severity and evolution of the disease^5^ with the possibility of hemorrhagic shock.^31^

Moreover, unreported pre-existing chronic comorbidities may have propitiated the hearing alterations, as exposed in the study by Diniz et al.,^19^ that included aseptic meningitis and acute kidney injury. The possibility of symptom remission and improvement in audibility reveals the importance of treatment and auditory monitoring, mainly because hearing alterations can happen late after DENV infection.^22^

CHIKV has been associated with decreased hearing acuity with persistent auditory symptoms,^7^ with sensory sensorineural hearing loss being of lower occurrence when compared to the other arboviruses studied here.^25^ The neurotropic nature of CHIKV affects auditory neurons, similarly to other viral infections, which affect the organ of Corti, vascular stria, and tectory membrane,^32^ enabling demyelination neuropathy and various auditory disorders in the infected.^33^, ^34^

In ZIKV infection, auditory alterations are specific manifestations that can occur during acute infection, having the character of transient sensory-neural impairment of gradual spontaneous resolution.^12^ Vinhaes and colleagues^13^ performed serial audiometry and showed that the sensory hearing loss had a transient character with an improvement of the audibility levels in up to 28 days.

The molecular and morphological damage to cochlear structures by ZIKV infection has been explained by multiple complex mechanisms that contribute to hearing loss;^35^ however, damage to central auditory pathways is questioned due to the neurotropic behavior of the virus.^36^, ^37^, ^38^

Because ZIKV is cytopathic to neurons, it infects microvascular endothelial cells in the brain, allowing viral access by impairing nuclear responses of innate immunity.^36^ In addition to disrupting the activity of essential proteins involved in developing the neurosensory system, such as ZPR1, the infection plays an evasive role in mediated dysregulation.^37^

A study^38^ using mice demonstrated that ZIKV infection in adult neural stem cells leads to cell death and reduced proliferation. These data suggested that adult neural stem cells are vulnerable to ZIKV neuropathology, just as the adult brain can be. This finding has been confirmed by studies,^39^, ^40^, ^41^ in humans demonstrating cranial nerve involvement causing severe encephalitis and other rare neurological disorders, vulnerability observed in adult neural cells to ZIKV neuropathology may generate consequences of exposure in the adult brain of late manifestation.

Neurological complications related to CHIKV and ZIKV arboviruses have been reported only in the most severe cases of arboviral infection or coinfection, demonstrating a rare viral neuropathic effect that is still poorly understood.^29^ CHIKV encephalitis presented as a brainstem syndrome and boomerang sign^24^ and ZIKV-associated neuromyelitis optical disorder,^22^ illustrate this scenario and show that both may be one of the causes of demyelination or tissue alteration post- or parainfectious.^22^, ^24^

Inflammatory processes with demyelinating lesions in the cochlear nerve may cause hearing impairment^22^; however, no study has directly evaluated this possibility. An essential factor to be considered is the effect of antibiotics used in some studies to ameliorate the acute symptoms of arboviral infection,^19^ as they are notoriously known to induce multiple adverse reactions in the body, such as resistance to bacteria and irreversible or long-lasting hearing alterations.^42^ Therefore, it cannot be ruled out that part of the hearing difficulties presented are consequences of iatrogenic drug treatment^43^ added to the neurotropic activity of viruses.

The main limitation of this research concerns the small amount of data that exists to characterize the hearing alterations in each arbovirus. Secondarily, the lack of homogenization between samples and the lack of objective data for measuring hearing function in some studies^12^, ^18^, ^22^, ^24^^,^^25^ added to the low follow-up time of the studies^21^, ^25^ probably underestimates the true prevalence and impairs the quality of the data by the risk of bias. Furthermore, no study has directly examined the relationship between other complications, such as neurological and hearing impairment.^22^, ^24^ Thus, future epidemiological studies with representative populations and careful methodology when evaluating hearing loss may promote the confirmation and generalization of the results presented.

## Conclusion

Preliminary evidence supports those hearing alterations may occur during Dengue, Chikungunya, and Zika infections, with the variable occurrence of auditory symptoms and the presence of sensorineural hearing loss that may be permanent or transient. Otalgia, hypoacusis, vertigo/tinnitus, and tinnitus were the most common symptoms, and sensorineural hearing loss was more remarkable for patients exposed to the Zika virus.

Audiological follow-up and treatment are suggested to reduce the severity of long-term auditory viral sequelae. However, due to the presence of limitations related to study designs, mainly by the high number of case reports and methodological limitations, the actual effect of arboviruses on hearing may be substantially different from the estimation in this review.

Therefore, future studies should aim to establish the causal relationship between auditory alterations in larger samples affected by these arboviruses in order to define a clear mechanism that explains the auditory symptoms.

## Funding

No funding was received.

## Conflicts of interest

The authors declare no conflicts of interest.
