# Supplementary material for: Does a patient with acquired arbovirus infection have a hearing impairment? A scoping review of hearing changes in an adult with Dengue, Chikungunya, and Zika
Source: Braz J Otorhinolaryngol. 2023 Oct 11;90(1):101342. doi: 10.1016/j.bjorl.2023.101342 (PMC10598399; doi:10.1016/j.bjorl.2023.101342)
Supplement: Supplementary file 1 [file mmc1.docx]

**BJORL-D-23-00024_Supplementary Material**

**Supplementary Table 1** Search terms used for the review.

| **Database** | **Search strategy** |
| --- | --- |
| Embase | 1. 'Zika virus'/exp OR 'zika virus' OR 'zika virus infection'/exp OR 'zika virus infection' OR 'chikungunya'/exp OR chikungunya OR 'chikungunya virus'/exp OR 'chikungunya virus' OR 'dengue'/exp OR dengue OR 'dengue virus'/exp OR 'dengue virus' OR 'Severe Dengue' OR 'severe dengue'/exp OR 'severe dengue' |
|  | 2. 'Zika' OR 'zikv'/exp OR zikv OR CHIKF OR CHIKV OR 'denv'/exp OR denv |
|  | 3. 1 OR 2 |
|  | 4. 'hearing'/exp OR 'hearing' OR 'hearing loss'/exp OR 'hearing loss' OR 'deafness'/exp OR 'deafness' OR 'hearing disorders'/exp OR 'hearing disorders' OR 'retrocochlear diseases'/exp OR 'retrocochlear diseases' OR 'auditory perceptual disorders'/exp OR 'auditory perceptual disorders' OR 'vestibulocochlear nerve diseases'/exp OR 'vestibulocochlear nerve diseases' |
|  | 5. 'audition'/exp OR 'audition' OR 'hypoacusis'/exp OR 'hypoacusis' OR 'hearing impairment'/exp OR 'hearing impairment' OR 'hearing disease' OR 'dysacusis' |
|  | 6. 4 OR 5 |
|  | 7. 3 AND 6 |
| PubMed | 1. "Zika Virus"[Mesh] OR "Zika Virus Infection"[Mesh] OR “Chikungunya Fever"[Mesh] OR "Chikungunya virus"[Mesh] OR "Dengue"[Mesh] OR "Dengue Virus"[Mesh] OR "Severe Dengue"[Mesh] |
|  | 2. Zika OR ZikV OR CHIKF OR CHIKV OR DENV |
|  | 3. |
|  | 4. "Hearing"[Mesh] OR "Hearing Loss"[Mesh] OR "Deafness"[Mesh] OR "Hearing Disorders"[Mesh] OR "Retrocochlear Diseases"[Mesh] OR "Auditory Perceptual Disorders"[Mesh] OR "Vestibulocochlear Nerve Diseases"[Mesh] |
|  | 5. Audition OR Hypoacusis OR "Hearing Impairment" OR "Hearing disease" OR Dysacusis |
|  | 6. 4 OR 5 |
|  | 7. 3 AND 6 |
| ScienceDirect | Title, abstract, keywords: ("zika virus" OR "Chikungunya virus" OR "Dengue virus" OR Dengue) AND (hearing OR "hearing loss" OR deafness OR "hearing disorders" OR "auditory perceptual disorders") |
| Scopus | TITLE‒ABS‒KEY (( "zika virus" OR "zika virus infection" OR "Chikungunya Fever" OR "Chikungunya virus" OR "Dengue" OR "Dengue Virus" OR "Severe Dengue" OR Zika OR ZikV OR CHIKF OR CHIKV OR DENV)) AND TITLE‒ABS‒KEY (( "hearing" OR "hearing loss" OR "deafness" OR "hearing disorders" OR "retrocochlear diseases" OR "auditory perceptual disorders" OR "vestibulocochlear nerve diseases" OR audition OR hypoacusis OR "hearing impairment" OR "hearing disease" OR dysacusis )) |
| Web of Science | (ALL=("Zika Virus" OR "Zika Virus Infection" OR “Chikungunya Fever" OR "Chikungunya virus" OR "Dengue" OR "Dengue Virus" OR "Severe Dengue" OR Zika OR ZikV OR CHIKF OR CHIKV OR DENV )) AND ALL=("Hearing" OR "Hearing Loss"OR "Deafness" OR "Hearing Disorders" OR "Retrocochlear Diseases" OR "Auditory Perceptual Disorders" OR "Vestibulocochlear Nerve Diseases" OR Audition OR hypoacusia OR "Hearing Impairment" OR "Hearing disease" OR dysacusia) |

**Supplementary Table 2** Change in degree of hearing loss considering four-frequency pure-tone average (4fPTA) of the thresholds at 500, 1000, 2000, and 4000 Hz for each ear, as suggested by the World Health Organization.[17]

| **Autor** | **Follow-up period** | **Inicial four-frequency pure-tone average** | | **Subsequent four-frequency pure-tone average** | | **Initial degree of hearing loss** | | **Degree of subsequent hearing loss** | | **Change in the degree of hearing loss** |
| --- | --- | --- | --- | --- | --- | --- | --- | --- | --- | --- |
|  |  | **4fPTA of right ear** | **4fPTA of left ear** | **4fPTA of right ear** | **4fPTA of left ear** | **Right ear** | **Left ear** | **Right ear** | **Left ear** |  |
| Denis et al., 2003 | Unclear | NR | NR | NR | NR | NR | NR | NR | NR | NR |
| Diniz et al., 2021 | Six-month | 57.5 | Abs | 26.25 | Abs | Moderately severe | Profound | Mild | Profound | Yes |
| Mughal et al., 2022 | Tree-month | 10 | 96.25 | 12.5 | 68.75 | Normal | Profound | Normal | Severe | Yes |
| Rahme et al., 2020^a^ | One month | 28.75 | Abs | 17.5 | 37.5 | Mild | Profound | Normal | Moderate | Yes |
| Ribeiro et al., 2015 | Seven-month | 120 | 100 | 120 | 100 | Profound | Profound | Profound | Profound | No |
| Soni et al., 2021 | Tree-month | NR | NR | NR | NR | NR | NR | NR | NR | NR |
| Couturier et al., 2012 | Two years | NR | NR | NR | NR | NR | NR | NR | NR | NR |
| Dutta et al., 2011 | Tree to six-month | NR | NR | NR | NR | NR | NR | NR | NR | NR |
| Jain et al., 2018 | Unclear | NR | NR | NR | NR | NR | NR | NR | NR | NR |
| Aspahan et al., 2019 | Unclear | NR | NR | NR | NR | NR | NR | NR | NR | NR |
| Martins et al.,2017 | Unclear | NR | NR | NR | NR | NR | NR | NR | NR | NR |
| Tappe et al., 2015 | Unclear | NR | NR | NR | NR | NR | NR | NR | NR | NR |
| Vinhaes et al., 2016^b^ | One month | 74.37 | 90 | 46.25 | 62.5 | Severe | Profound | Moderate | Moderately severe | Yes |

NR, Not Reported; Abs, Absent.

^a^ Thresholds provided only from one of the patients.

^b^ Average of the thresholds of the two patients who presented hearing loss.
